# Genome-Wide Identification of the SRZ Gene Family in Rice and *Arabidopsis*, and Functional Analysis of OsSRZ1 in Regulating Panicle Exsertion

**DOI:** 10.3390/biology15161346

**Published:** 2026-08-08

**Authors:** Chao Zhang, Sitian Fei, Yingxiang Hou, Jingjing Xu, Qiannan Sun, Chaofeng Shen, Siyu Liu, Lan Li, Yong Luo

**Affiliations:** 1 Nanling Research Institute for Modern Seed Industry, Xiangnan University, Chenzhou 423000, China; kingofzc@126.com (C.Z.); feisitian@xnu.edu.cn (S.F.); houyingxiang@xnu.edu.cn (Y.H.); lilan7@hnu.edu.cn (L.L.); 2 Chenzhou Industrial Technology Research Institute, Xiangnan University, Chenzhou 423000, China; siruner@163.com; 3 Hunan Provincial Key Laboratory of the Traditional Chinese Medicine Agricultural Biogenomics, Changsha Medical University, Changsha 410219, China; 13574368135@163.com; 4 Chenzhou Institute of Agricultural Sciences, Chenzhou 423000, China; sqiann@126.com (Q.S.); scfawsa@126.com (C.S.); 5 School of Pharmacy, Xiangnan University, Chenzhou 423000, China; 6 School of Chemistry and Environmental Science, Xiangnan University, Chenzhou 423000, China

**Keywords:** SRZ family, plant height, panicle exsertion, OsSRZ1, PCD, CRISPR/Cas9

## Abstract

The Stress-Repressive Zinc Finger (SRZ) family is a class of zinc finger proteins in plants that has been closely associated with plant growth, development, and stress responses. In this study, we conducted a genome-wide identification of the SRZ family members and identified 14 and 10 members in rice and *Arabidopsis*, respectively. We further characterized their phylogenetic relationships, gene structures, conserved motifs, promoter cis-elements, synteny, and spatiotemporal expression patterns. To investigate the functional role of this family in rice, we generated targeted mutants of *OsSRZ1* using the CRISPR/Cas9 system. Phenotypic analysis revealed that *srz1* knockout mutants exhibited significantly reduced plant height. Notably, the homozygous mutant *srz1* completely failed in panicle exsertion from the flag leaf sheath, resulting in complete enclosure, whereas the heterozygous mutant *srz1(h)* exhibited only partial panicle exsertion. Further cytological observations revealed abnormal floral organ development and premature anther senescence in *srz1*, accompanied by a significant reduction in seed setting rate—*srz1(h)* set significantly fewer seeds, whereas *srz1* exhibited complete sterility with a seed setting rate of 0%. Collectively, these findings suggest that members of the SRZ family may play important roles in rice growth and development. Our results demonstrate that OsSRZ1 is a critical regulator of plant height and panicle exsertion by regulating internode elongation, and that its disruption leads to premature anther programmed cell death (PCD) and consequent seed setting failure.

## Simple Summary

Rice feeds more than half of the world’s people, so maintaining its high yield is critical for global food security. A key factor for yield is the successful emergence of the rice panicle from the leaf sheath; failure in this process drastically reduces seed production. However, the genes that control this process are not well understood. In this study, we first systematically identified all members of the *SRZ* gene family in both rice and *Arabidopsis* at the genome-wide level. This comprehensive identification provides a foundational list of candidate genes for future research. We then focused on one of these genes in rice, *OsSRZ1*, and demonstrated that its mutation severely blocks panicle emergence, shortens the panicle, and lowers the seed setting rate. Our work not only reveals a key player in panicle development, but also provides a valuable genetic resource and targets for breeding programs aimed at improving rice plant architecture and yield, which is essential for feeding a growing global population.

## 1. Introduction

Over the past two decades, advances in sequencing and bioinformatics have enabled systematic characterization of numerous plant gene families, greatly facilitating functional genomic studies [1]. Numerous gene families have been extensively identified across plant species, and their functions are being progressively revealed. Zinc finger proteins (ZFPs) contain one or more conserved ZnF domains—relatively small protein motifs with multiple finger-like protrusions that enable tandem contacts with target molecules [2,3]. Through these domains, most ZFPs mediate interactions with DNA, RNA, proteins, and even lipids [4,5,6]. As one of the largest protein families in plants, ZFPs are known to participate in a wide range of biological processes, encompassing transcriptional regulation, post-transcriptional control, RNA metabolism, chromatin remodeling, cell cycle progression, DNA repair, protein folding and turnover, cytoskeletal dynamics, hormone signaling, and responses to biotic and abiotic stresses [7,8,9,10,11]. Based on the number and arrangement of conserved cysteine (Cys) and histidine (His) residues, ZFPs can be classified into multiple subfamilies, including the classical C2H2-type, C2HC-type, CCCH-type, and others. Among them, the stress-repressive zinc finger (SRZ) proteins constitute a distinct and poorly characterized group defined by the atypical GDWXCX_2−4_NX_6_CX_2_C motif (where X denotes any amino acid), which belongs to the RanBP2-type zinc finger family. Unlike the C2H2-type zinc fingers that function as DNA-binding transcription factors, RanBP2-type ZnF domains are characterized by a distinct structural fold and, in mammals, serve as components of the nuclear pore complex mediating nucleocytoplasmic transport through binding to RanGDP [12,13]. In the present study, we adopt the unified designation ‘SRZ’ (Stress-Repressive Zinc Finger) for this family, following the initial identification and naming of the first rice member, OsSRZ1 (stress-repressive zinc finger protein 1), as reported by Huang et al. [14]. This functional divergence suggests that SRZ proteins may function beyond the conventional roles of typical zinc finger proteins. However, their biological functions in plants remain largely unexplored.

RNA-binding proteins (RBPs) are characterized by their ability to associate with RNA molecules through diverse RNA-binding domains (RBDs), thereby governing nearly every aspect of post-transcriptional regulation—from RNA processing and stability to translation and turnover. In *Arabidopsis*, the CCCH zinc finger protein HUA1 and the KH domain-containing protein HEN4 physically interact to promote AGAMOUS pre-mRNA processing, thereby specifying stamen and carpel identities [15]. In addition, two RRM-containing proteins, AtRBP45a and AtRBP45c, are specifically expressed in anthers and are presumed to mediate translational repression during pollen maturation [16]. In rice, the RNA-binding protein OsRRM1 interacts with the orphan protein OsFON879 to stabilize *OsMADS1* transcripts and regulate floral organ homeostasis [17]. OsDMCK1 is essential for male fertility by regulating meiotic cytokinesis [18], and another rice RNA-binding protein, OsLa1, influences grain length and pollen fertility by binding to *OseIF6.1* mRNA, while its homolog OsLa2 affects grain weight and seed-setting rate [19]. Collectively, these examples illustrate that post-transcriptional regulation by RNA-binding proteins is an essential layer of control in flower and reproductive development. Despite these advances, the functional roles of many RBP families, including the SRZ family in rice, remain largely unexplored.

Recent studies have begun to reveal the functional importance of RanBP2-type zinc finger proteins in plants. In *Arabidopsis*, *JUL1* (*AtSRZ1*, same locus ID) and its homolog *JUL2* (*AtSRZ2*) encode RanBP2-type zinc finger RNA-binding proteins. They redundantly repress phloem development by translationally inhibiting SMXL4/5 via binding to G-quadruplex structures in their 5′ UTRs [20,21]. Notably, JUL1 modulates leaf adaxial–abaxial polarity by associating with pri-MIR165a, blocking DCL1-mediated processing, and reducing miR165/166 abundance to regulate the miR165/166-HD-ZIP III module. Though JUL1 is vasculature-specific, its protein moves to adjacent tissues as a non-cell-autonomous signal. JUL2 shares the phloem function and may mediate light-dependent root growth signaling [20,21]. SRP1 (AtSRZ5) is an RNA-binding protein repressed by ABA, salt and cold. It negatively modulates ABA signaling by binding the 3′ UTR of ABI2 to suppress its expression [22]; *srp1* knockout and overexpression lines show attenuated and enhanced ABA/salt sensitivity, respectively. OZ1 (AtSRZ9) is critical for chloroplast RNA editing via its zinc finger and C-terminal domains; *oz1* mutants show variegated leaves, retarded chloroplast growth and reduced palisade cells [13,23,24]. SED1 (AtSRZ6) is a mitochondrial protein essential for embryogenesis; *sed1* mutants exhibit defective embryogenesis with abnormal mitochondria and plastids, leading to embryo abortion [25]. TAF15b (AtSRZ10) promotes flowering by repressing *FLC* transcription through Pol II dephosphorylation; *taf15b* mutants show late flowering with elevated *FLC*, fully rescued in *flc taf15b* double mutants. Moreover, TAF15b localizes to nuclei and cytoplasmic processing bodies and regulates plant immunity by modulating SNC1 accumulation post-transcriptionally [26,27,28].

Compared with *Arabidopsis*, functional characterization of *SRZ* genes in rice remains far less advanced. To date, only a few rice SRZ members have been functionally investigated. *OsSRZ1* encodes a RanBP2-type zinc finger protein. Its transcript levels are downregulated by various abiotic stresses, and its ectopic expression in tobacco enhances sensitivity to cold and salt stress, suggesting a negative role in stress signaling [14]. Additionally, *OsSRZ1* was found to be up-regulated in response to ABA, JA, and H_2_O_2_ treatments [29]. Collectively, these observations indicate that OsSRZ1 and its homologs may function as negative regulators in abiotic stress responses, and their transcriptional regulation might be crucial for plant stress tolerance. Loss-of-function of *OsLS1* (*OsSRZ1*) leads to premature leaf senescence, accompanied by increased ROS accumulation and accelerated programmed cell death (PCD) [30]. OsRZF1 (OsSRZ3) functions as a key regulator of Mg homeostasis in rice. The *lmgc1* mutant, caused by disruption of *OsRZF1*, exhibits reduced Mg^2+^ influx in roots and impaired root-to-shoot Mg translocation [31]. *OsSTL1* (*OsSRZ4*) was identified as a candidate gene for salt tolerance through GWAS, and its superior haplotype contains a functional SNP likely contributing to salt tolerance [32]. Collectively, these findings indicate that RanBP2-type zinc finger proteins in plants participate in diverse biological processes. Nevertheless, the regulatory networks and detailed molecular mechanisms of most SRZ proteins remain mostly elusive and merit further investigation.

In this study, we systematically characterized the *SRZ* gene family in rice and *Arabidopsis* through integrative genomic and phylogenetic analyses, and further investigated the functional role of *OsSRZ1* using a CRISPR/Cas9-generated knockout mutant. Our results demonstrate that OsSRZ1 is a pleiotropic regulator of rice growth and reproduction, controlling both internode elongation and seed-setting rate. Loss of *OsSRZ1* function reduced plant height and panicle exsertion, and caused premature anther PCD and pollen abortion, ultimately leading to severe decrease in seed-setting.

## 2. Materials and Methods

### 2.1. Identification of SRZ Proteins in Arabidopsis and Rice

To identify SRZ members in *Arabidopsis* and rice, BLAST searches were performed against the EnsemblPlants (https://plants.ensembl.org) using four known rice SRZ sequences [14] as queries. Subsequently, the obtained sequences were submitted to the SMART database (http://smart.embl.de/smart/change_mode.cgi (accessed on 13 July 2026)) [33] to confirm the presence of conserved GDWXCX2-4NX6CX2C motifs characteristic of the SRZ family.

### 2.2. Phylogenetic and Synteny Analysis in Rice and Arabidopsis

To investigate the evolutionary relationships and genomic conservation of the SRZ family between rice and *Arabidopsis*, multiple sequence alignments and phylogenetic analyses were performed using MEGA6.0 [34] with the neighbor-joining (NJ) method, p-distance, pairwise deletion of gaps, and default assumptions, and node support was tested with 1000 bootstrap replicates. For synteny analysis, chromosomal locations of all identified SRZ genes were retrieved from the corresponding genome annotation files, and inter-species syntenic relationships were analyzed and visualized using TBtools (v2.485) [35] with the MCScanX algorithm and the Multiple Synteny Plotter module.

### 2.3. Gene Structure and Motif Organization Analysis in Arabidopsis and Rice

Exon–intron structures of the *SRZ* genes were generated using the Gene Structure View (Advanced) module of TBtools (v2.485) [35]. Briefly, the genome annotation file (GFF3) was imported into TBtools (v2.485), and the gene structure diagrams were automatically retrieved and displayed based on the provided *SRZ* gene ID list. To identify conserved domains, the full-length amino acid sequences of SRZ proteins were submitted to the SMART database (http://smart.embl.de/smart/change_mode.cgi (accessed on 13 July 2026)) [33] for motif identification.

### 2.4. Cis-Element Prediction of SRZ Gene Promoters in Rice and Arabidopsis

To identify putative cis-regulatory elements in the promoter regions of *SRZ* genes, the 2000-bp genomic sequences upstream of the translational start codons were submitted to the PlantCARE database (http://bioinformatics.psb.ugent.be/webtools/plantcare/html/ (accessed on 13 July 2026)) for promoter analysis.

### 2.5. Spatiotemporal Expression Patterns of SRZ Genes in Rice

Microarray-based spatio-temporal expression profiles of *OsSRZs* in various tissues and at different developmental stages were analyzed using publicly available microarray data. The expression data were retrieved from the RXP_0001 dataset in the Rice Expression Profile Database (https://rapdb.dna.naro.go.jp/ (accessed on 13 July 2026)). Heatmaps were generated with R (Version 2026.05.0+218; Posit Software, PBC (Boston, MA, USA), 2026).

### 2.6. Construction of the CRISPR/Cas9 Editing Vector

The CRISPR/Cas9 genome-editing system has been widely used for targeted gene mutagenesis in plants. In this study, the binary vector pYLCRISPR/Cas9 and the gRNA expression cassettes used for vector construction were provided by the team of Prof. Yaoguang Liu at South China Agricultural University. We constructed the editing vector following the protocol described by Ma et al. [36]. For targeted mutagenesis of the *SRZ1* gene, two target sequences of 20 bases immediately upstream of the PAM motifs were selected and designated as T1 (AGCCAGGAGACTGGGACTGCAGG) and T2 (TCTATGTTTGATCACAGGTCTGG). The corresponding sgRNAs were individually cloned into the pYLsgRNA-U3 and pYLsgRNA-U6a expression cassettes, in which transcription of the sgRNAs is driven by the rice U3 and U6a promoters, respectively. The two sgRNA expression cassettes were then assembled into the pYLCRISPR/Cas9 binary vector via overlapping PCR using the universal primers F-U/R-gRNA and the site-specific primer pairs F-B1/R-B2 and F-B2/R-BL. The resulting recombinant plasmid, designated pYLCRISPR/Cas9-*SRZ1*-T1/T2, carries the hygromycin B phosphotransferase (HPT) gene as a selectable marker for transgenic plant screening. All primers used for vector construction are listed in Table 1.

### 2.7. Rice Transformation and Generation of srz1 Mutant Lines

The recombinant plasmid pYLCRISPR/Cas9-*SRZ1*-T1/T2 was introduced into Agrobacterium tumefaciens strain EHA105 via electroporation, and positive colonies were selected for subsequent transformation. Rice transformation was performed using embryogenic calli derived from mature seeds of the rice variety R893 as explants, as previously described [37]. Briefly, the embryogenic calli were immersed in the Agrobacterium suspension (OD_600_ = 0.1–0.3) for 15 min, blotted dry on sterile filter paper, and then co-cultivated on co-cultivation medium at 28 °C in the dark for 2–3 days. Following co-cultivation, the calli were thoroughly washed with sterile water containing 500 mg/L cefradine and then transferred to selection medium supplemented with 500 mg/L cefradine and 40 mg/L hygromycin B. After 3–4 weeks of selection, hygromycin-resistant calli began to proliferate, whereas non-transformed calli turned brown and gradually died. The resistant calli were subcultured on fresh selection medium every 3 weeks for two to three rounds to enrich for stably transformed callus lines. For shoot differentiation, the resistant calli were transferred to regeneration medium containing 0.5 mg/L 6-BA and 0.2 mg/L NAA. Bud differentiation was initiated after 4–6 weeks, and shoots developed rapidly with emerging leaves. When the regenerated shoots reached 3–5 cm in height, they were excised and transferred to rooting medium (half-strength Murashige and Skoog medium) for root development. After 4–6 weeks, whole plantlets with well-developed roots were acclimatized and transplanted to soil in a greenhouse for further growth.

To identify CRISPR/Cas9-induced mutations at the target sites, genomic DNA was extracted from leaf tissues of transgenic plants using a Plant Genomic DNA Kit (TsingKe Biotech, Beijing, China). PCR amplification was performed using gene-specific primers *SRZ1*-F and *SRZ1*-R (Table 1) flanking the two target sites, yielding an amplicon of 858 bp. The purified PCR products were subjected to Sanger sequencing, and the sequencing chromatograms were visually analyzed using Chromas software (2.2.3) to determine the mutation types and zygosity of each transgenic line. Sequence alignments were performed using MEGA software (version 6.0, https://www.megasoftware.net/) to confirm the mutations at the target sites. The homozygous and heterozygous mutants were designated as *srz1* and *srz1(h)*, respectively.

### 2.8. Statistical Analysis of Agronomic Traits

All agronomic traits were measured and statistically analyzed under normal field growth and management conditions. under normal field growth and management conditions. All phenotypic analyses were performed using T1 and T2 generation plants. Since the homozygous *srz1* mutants were completely sterile and unable to set seeds, seeds of *srz1* were obtained from *srz1(h)* plants in each generation and grown for subsequent analyses. The agronomic traits investigated included plant height, panicle length, number of grains per panicle, and seed setting rate. Seed setting rate was calculated using the formula Seed Setting Rate (%) = (Number of Filled Grains/Total Number of Spikelets) × 100, where total spikelets include both filled and unfilled grains. For each genotype, the data are presented as mean ± SD. Statistical analyses and graph preparation were performed using GraphPad Prism (version 8.0.1). Statistical significance between groups was determined using Student’s *t*-test. A probability value of *p* < 0.05 was considered statistically significant.

### 2.9. Pollen Fertility Assay

To evaluate pollen fertility, mature anthers were collected from R893 and *srz1* plants at the heading stage. Pollen grains were stained with 1% iodine–potassium iodide (I_2_-KI) solution and examined under a light microscope. Fully stained, dark-brown pollen grains were considered fertile, while unstained or irregularly shaped pollen grains were scored as sterile.

### 2.10. TUNEL Assay

To investigate whether programmed cell death (PCD) is involved in the anther abortion of *srz1* mutants, TUNEL staining was conducted on anther sections. Anthers harvested at the heading stage were fixed in FAA solution, dehydrated through an ethanol series, and embedded in paraffin wax [38]. TUNEL labeling was performed using a DeadEnd Fluorometric TUNEL system (Promega, #G3250, Madison, WI, USA) according to the manufacturer’s instructions.

## 3. Results

### 3.1. Identification of SRZ Proteins in Rice and Arabidopsis

To identify SRZ family members in rice and *Arabidopsis*, BLASTP searches were performed against the EnsemblPlants database using four known rice SRZ proteins as queries. Candidate sequences were further verified by SMART domain analysis and the conserved GDWXCX_2−4_NX_6_CX_2_C motif. A total of 14 *SRZ* genes were identified in rice and 10 in *Arabidopsis*, named as *OsSRZ1*–*OsSRZ14* and *AtSRZ1*–*AtSRZ10*, respectively. The detailed information for each SRZ is presented in Table 1, including gene name, RGAP ID, chromosome location, length of protein, and function. As summarized in Table 2, the protein lengths of SRZ members varied considerably, ranging from 138 amino acids (OsSRZ3) to 950 amino acids (OsSRZ6), with an average length of approximately 414 amino acids.

### 3.2. Phylogenetic Relationships and Synteny of SRZ in Rice and Arabidopsis

**Figure 1:**
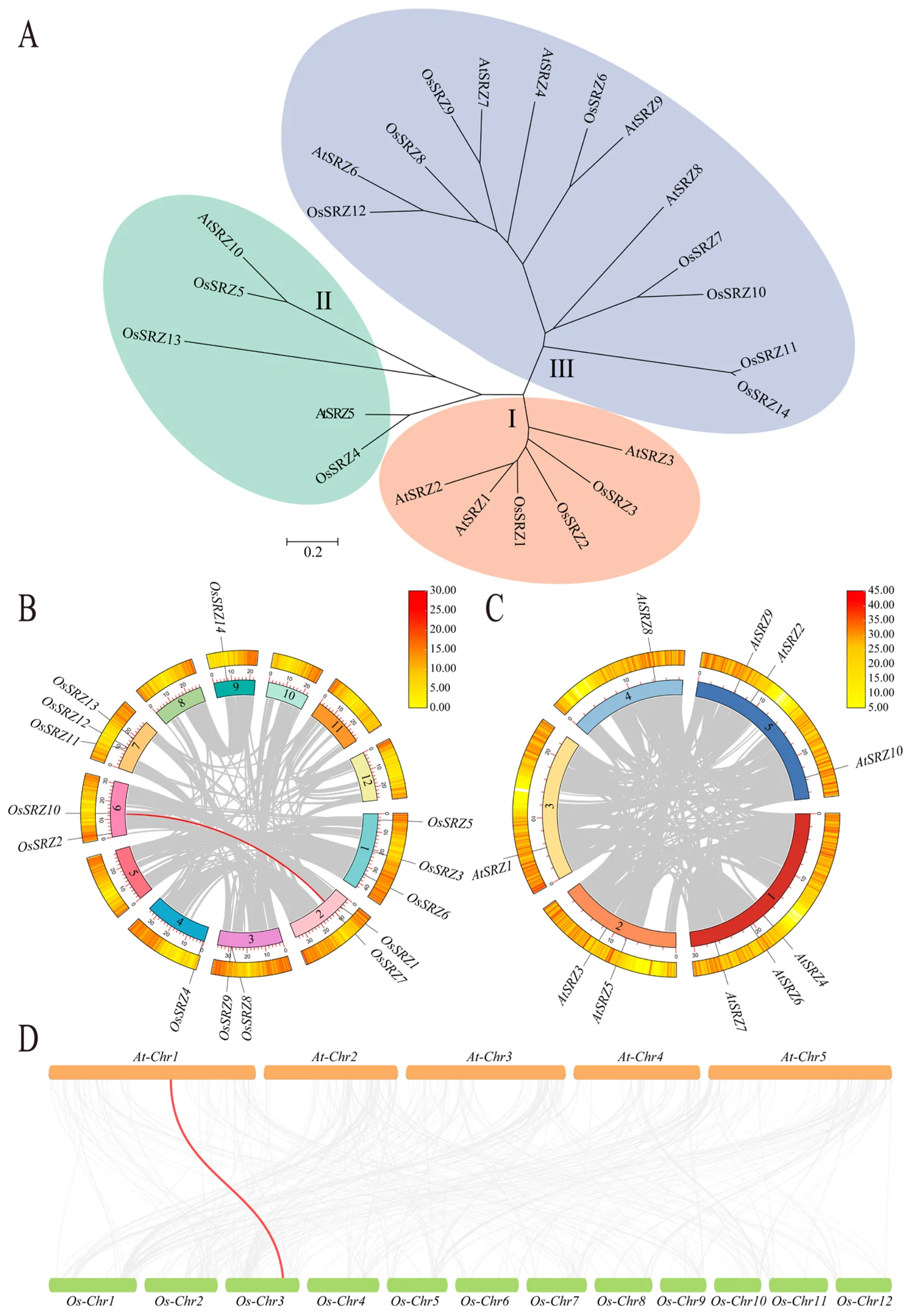
Phylogenetic relationships, chromosomal distribution, and synteny analysis of the *SRZ* gene family in rice and *Arabidopsis*. (**A**) Phylogenetic tree of SRZ proteins from rice and *Arabidopsis*. Colored branches and labels indicate three distinct clades (Groups I–III). The species origins of the genes are *O. sativa* (Os) and *A. thaliana* (At). (**B**) Circos plot showing the chromosomal distribution of *SRZ* genes in rice. (**C**) Circos plot showing the chromosomal distribution of *SRZ* genes in *Arabidopsis*. (**D**) Interspecies synteny dot plot between rice and *Arabidopsis*. The gray background lines represent all collinearity modules, and the red lines represent collinear gene pairs.

To investigate the evolutionary dynamics and expansion mechanisms of the SRZ gene family, we constructed a phylogenetic tree using full-length protein sequences of all 24 SRZ members and performed synteny analysis to identify duplication events and examine interspecies collinearity. The phylogenetic tree revealed that the 24 SRZ proteins were classified into three distinct clades (Groups I–III), with OsSRZ and AtSRZ members interspersed across all groups (Figure 1A). Group I contained six members, with a balanced distribution of three from each species (OsSRZ1–OsSRZ3 and AtSRZ1–AtSRZ3). Group II comprised five members, including three rice (OsSRZ4, OsSRZ5, and OsSRZ13) and two *Arabidopsis* (AtSRZ5 and AtSRZ10) proteins. The remaining 13 members formed the largest clade (Group III), with eight from rice and five from *Arabidopsis*.

In the rice Circos plot, only one syntenic gene pair (*OsSRZ7* and *OsSRZ10*) was identified (Figure 1B), implying that segmental duplication contributes minimally to the expansion of the rice *SRZ* gene family. Notably, these two genes form the closest sister clade in the phylogenetic tree, which further suggests that they may share partial functional redundancy derived from their shared evolutionary origin. Additionally, *OsSRZ8* and *OsSRZ9* were found to be closely positioned on chromosome 3, with an intergenic distance of approximately 181 kb (positions 28,588,615–28,593,698 and 28,774,588–28,777,248, respectively). This close chromosomal proximity, together with their clustering within the same clade (Group III) (Figure 1A), suggests that these two genes may have arisen from a tandem duplication event. This notion is further supported by their highly similar protein features, including comparable lengths (504 vs. 523 aa) and each containing two copies of the conserved GDWXCX_2−4_NX_6_CX_2_C motif (Table 1). In contrast, the *Arabidopsis* Circos plot revealed no SRZ-containing syntenic blocks or tandemly duplicated gene pairs (Figure 1C). All ten *AtSRZ* genes are distributed as singletons across the five *Arabidopsis* chromosomes, suggesting that the *SRZ* gene family in *Arabidopsis* has undergone limited expansion through duplication events. In the interspecies synteny dot plot, *OsSRZ8* was found to form a collinear pair with *AtSRZ4* (Figure 1D), suggesting that these two genes may represent orthologous pairs retained within conserved syntenic blocks between rice and *Arabidopsis*.

### 3.3. Gene Structure and Motif Organization of SRZ Genes in Rice and Arabidopsis

**Figure 2:**
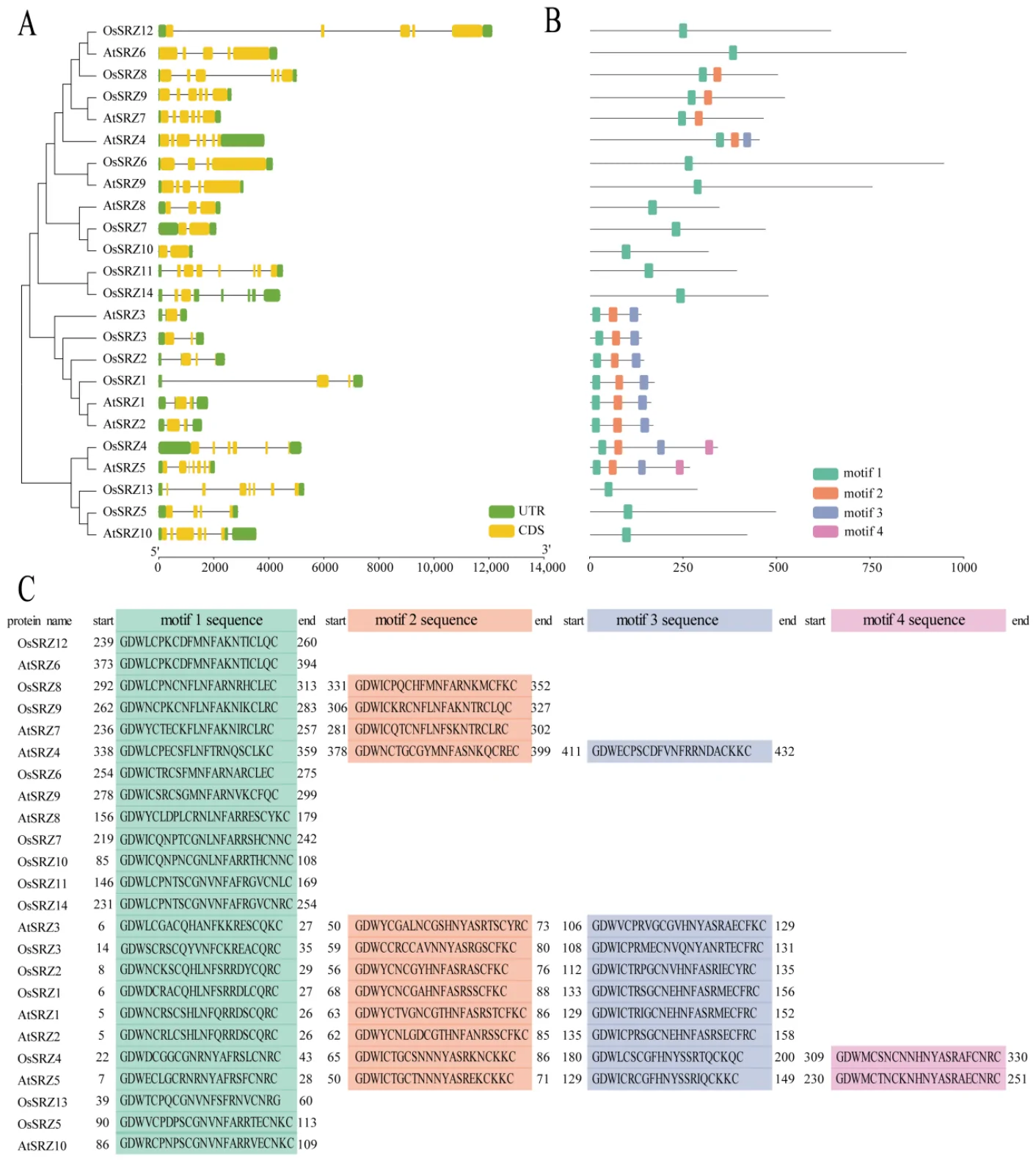
Gene structure and conserved motif organization of *SRZ* genes in rice and *Arabidopsis*. (**A**) Exon–intron structures of *SRZ* genes. Yellow boxes represent CDS (exons), green boxes represent untranslated regions (UTRs), and black lines represent introns. The phylogenetic tree on the left is based on the full-length protein sequences of SRZ members. (**B**) Schematic distribution of the conserved GDWXCX_2−4_NX_6_CX_2_C motif among SRZ proteins. Each colored box represents a single motif copy, with different colors indicating different motif variants. (**C**) Sequence alignment of the conserved GDWXCX_2−4_NX_6_CX_2_C motif copies from SRZ proteins. The full sequences of the motif copies are shown. The start and end positions of each conserved domain are also marked.

To gain further insights into the structural diversity of the *SRZ* gene family, we analyzed their exon–intron organization and conserved motif compositions. As shown in Figure 2A, the gene lengths varied considerably, ranging from 1035 bp (*AtSRZ3*) to 12,187 bp (*OsSRZ12*), with most genes being less than 8 kb in length. The exon numbers ranged from 2 to 8, with most genes containing 4–7 exons. Notably, *OsSRZ13* had the highest exon number (8), while *OsSRZ7*, *OsSRZ10*, *AtSRZ1*, *AtSRZ2*, and *AtSRZ3* had the lowest (2 exons each). Genes that clustered closely in the phylogenetic tree tended to share similar exon–intron structures. For instance, *OsSRZ7* and *OsSRZ10*, which exhibited highly similar gene structures, were the most closely related members in the phylogenetic analysis. Likewise, *AtSRZ7* showed a gene structure similar to that of *OsSRZ8* and *OsSRZ9*, and *AtSRZ4* clustered with *OsSRZ7* and *OsSRZ10*, all sharing a conserved structural framework in their first five exons. These observations suggest that exon–intron structural features are generally consistent with phylogenetic grouping, further supporting the evolutionary conservation of gene structure within this family.

To investigate the structural diversity of SRZ proteins, we analyzed the distribution of the conserved GDWXCX_2−4_NX_6_CX_2_C motif among the 24 SRZ members (Figure 2B). The number of motifs per protein ranged from 1 to 4, with 13 members containing two or more tandem repeats (3 members with two motifs, 8 with three motifs, and 2 with four motifs). Specifically, only OsSRZ4 and AtSRZ5 possessed four conserved motifs. As shown in Figure 2C, sequence alignment of the motif copies revealed that the GDWXCX_2−4_NX_6_CX_2_C motif was highly conserved, whereas the X residues exhibited considerable diversity. Notably, although these motifs were arranged in tandem, their sequences were not identical, with several amino acid variations observed among different copies. No obvious correlation was observed between motif number and protein length. For instance, OsSRZ6, the longest member (950 aa), contains only a single motif, whereas OsSRZ3, one of the shortest members (139 aa), harbors three conserved motifs. These results highlight the structural heterogeneity of SRZ family members, which may contribute to their functional specialization.

### 3.4. Spatio-Temporal Expression Patterns of SRZ Genes in Rice

**Figure 3:**
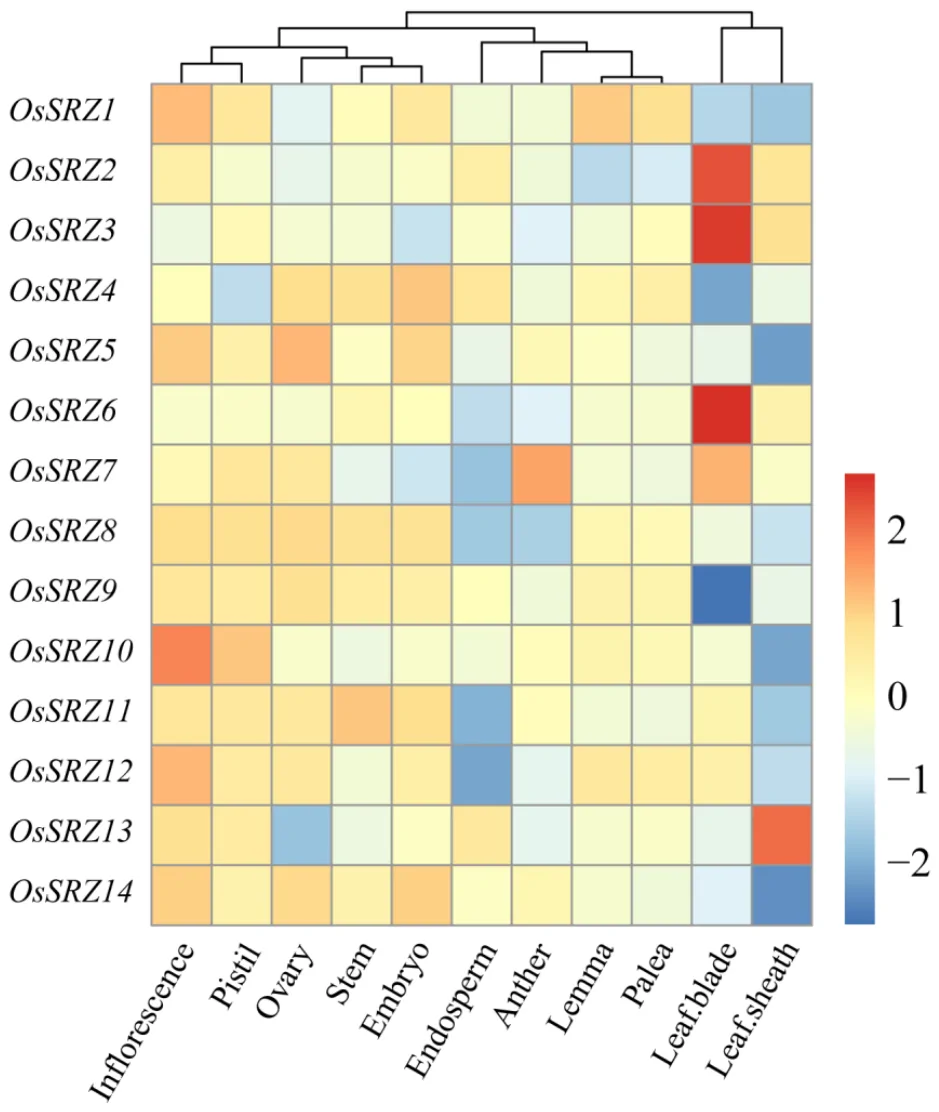
Spatiotemporal expression patterns of *SRZ* genes in rice. Heatmap showing the expression levels of *SRZ* genes across different tissues and developmental stages. The color scale represents relative expression levels, with red indicating high expression and blue indicating low expression.

To investigate the potential physiological functions of the *SRZ* gene family, we examined their spatio-temporal expression patterns across various rice tissues and developmental stages using publicly available microarray transcriptome datasets from the RiceXPro database (RXP_0001). As shown in the expression heatmap (Figure 3), the 14 *OsSRZ* members exhibited markedly distinct and tissue-preferential patterns across tissues from the vegetative stage (leaf blade and leaf sheath), reproductive stage (inflorescence, stem, pistil, ovary, anther, lemma, and palea), and mature stage (embryo and endosperm). Further analysis revealed that *OsSRZ1* showed its lowest transcript levels in vegetative tissues (leaf blade and leaf sheath), but was markedly expressed in reproductive organs, with the highest abundance in inflorescences, lemmas, and paleas, and moderate levels in stems and pistils. In contrast, *OsSRZ2*, *OsSRZ3*, and *OsSRZ6* shared similar expression profiles, characterized by dominant expression in leaf blades, moderate accumulation in leaf sheaths, and uniformly low expression in all other examined tissues. Notably, these three genes exhibited the highest transcript levels in leaf blades among all 14 *SRZ* genes, implying their potential functional specialization in photosynthetic tissues. Most *SRZ* genes displayed relatively low transcript levels in leaf sheaths, whereas *OsSRZ13* showed an opposite expression trend, with markedly higher transcript accumulation in leaf sheaths than in any other tested organs. In terms of temporal expression characteristics, the majority of *SRZ* genes were preferentially expressed at reproductive and mature stages rather than at the vegetative stage, with only five members (*OsSRZ2*, *OsSRZ3*, *OsSRZ6*, *OsSRZ7*, and *OsSRZ13*) exhibiting higher expression during vegetative growth. Such spatiotemporal expression patterns suggest that most *SRZ* members may perform their biological functions predominantly after the vegetative growth stage in rice.

### 3.5. Cis-Acting Element Analysis of SRZ Gene Promoters in Rice and Arabidopsis

**Figure 4:**
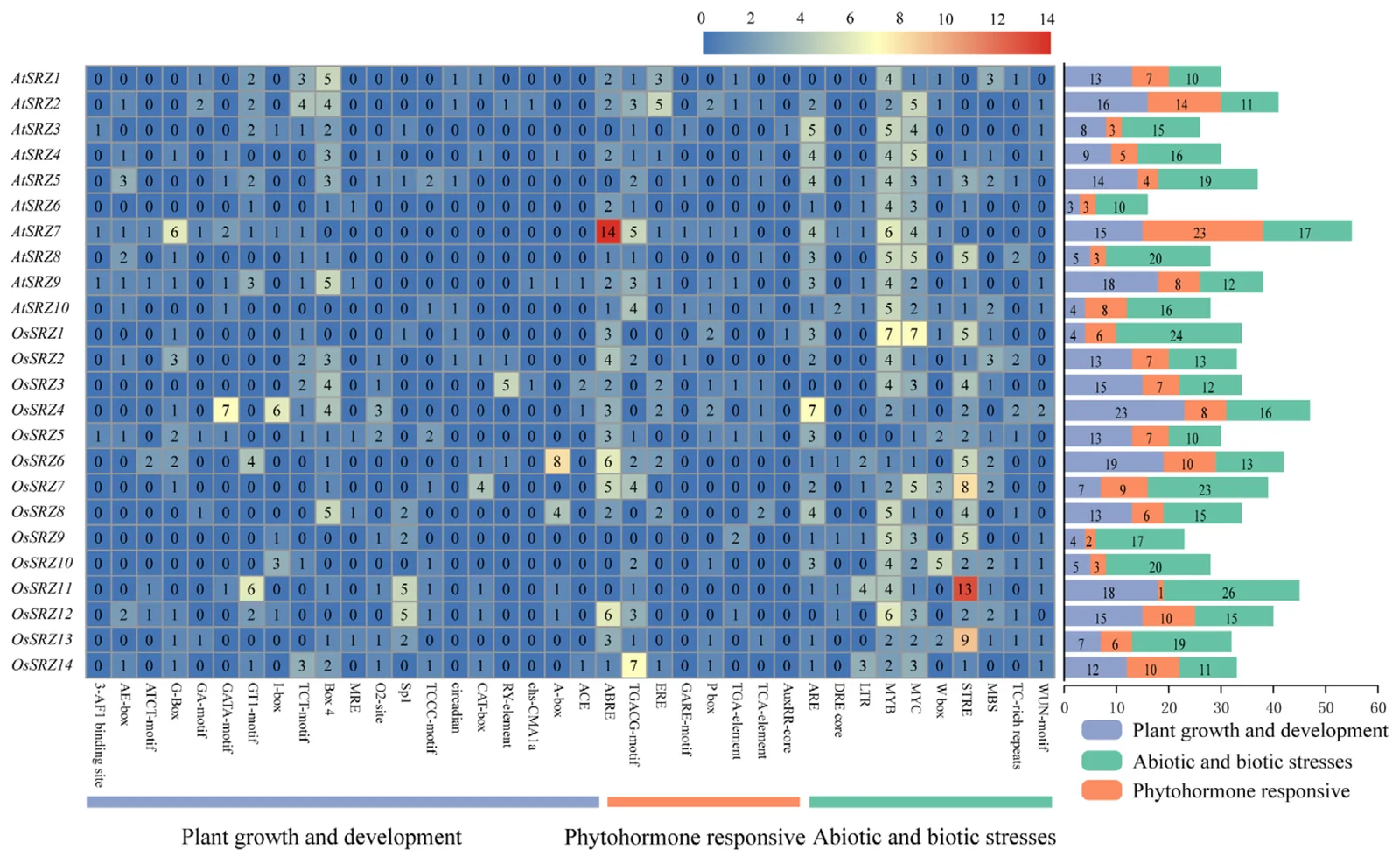
Cis-acting element analysis of *SRZ* gene promoters in rice and *Arabidopsis*. (**Left panel**) distribution of major cis-element types in the promoter regions of *SRZ* genes. (**Right panel**) numbers of cis-acting elements in the promoters of *SRZ* genes, classified into three major categories: abiotic and biotic stress-related elements, plant growth and development-related elements, and phytohormone-responsive elements. Gene names are shown on the left for both panels.

To further survey the transcriptional regulatory features of the *SRZ* gene family, we analyzed the cis-acting elements within the 2 kb sequences upstream of the ATG start codon of *SRZ* genes (Figure 4). A total of over 100 cis-element types were initially identified and subsequently categorized into three major classes based on their known functions: plant growth and development, phytohormone responsiveness, and abiotic and biotic stresses, along with additional elements with unknown functions. For subsequent analysis, we focused on approximately 40 major types for further analysis. The total number of cis-elements per promoter within the three major categories ranged from 16 (*AtSRZ6*) to 55 (*AtSRZ7*). The high count in *AtSRZ7* was attributed to its abundant phytohormone-responsive elements (23), along with 17 abiotic and biotic stress-related elements and 15 plant growth and development-related elements. In contrast, *AtSRZ6* had only 10 stress-related elements, 3 phytohormone-responsive elements, and 3 growth and development-related elements (Figure 4). Most *SRZ* genes had total element counts ranging from 20 to 30, whereas *AtSRZ6* (18) fell below 20, and six members (*OsSRZ12*, *AtSRZ2*, *OsSRZ6*, *OsSRZ11*, *OsSRZ4*, and *AtSRZ7*) exceeded 30 elements (Figure 4). Further analysis revealed that abiotic and biotic stress-related elements accounted for approximately one-third of the total cis-elements among the three major categories, mainly including MYB-binding sites, MYC-binding sites, stress response elements (STRE), and anaerobic induction elements (ARE). Most *SRZ* genes had fewer than 10 copies of any single cis-element type in their promoters, except for *AtSRZ7* with 14 abscisic acid-responsive elements (ABRE) and *OsSRZ11* with 13 STREs. Notably, some of these cis-elements overlap within the promoter sequences. Overall, the *SRZ* gene promoters harbored diverse cis-elements, with phytohormone-responsive and stress-related elements being particularly abundant, suggesting that they may be functionally associated with stress responses and hormone signaling pathways.

### 3.6. Role of OsSRZ1 in Regulating Plant Height and Internode Elongation in Rice

**Figure 5:**
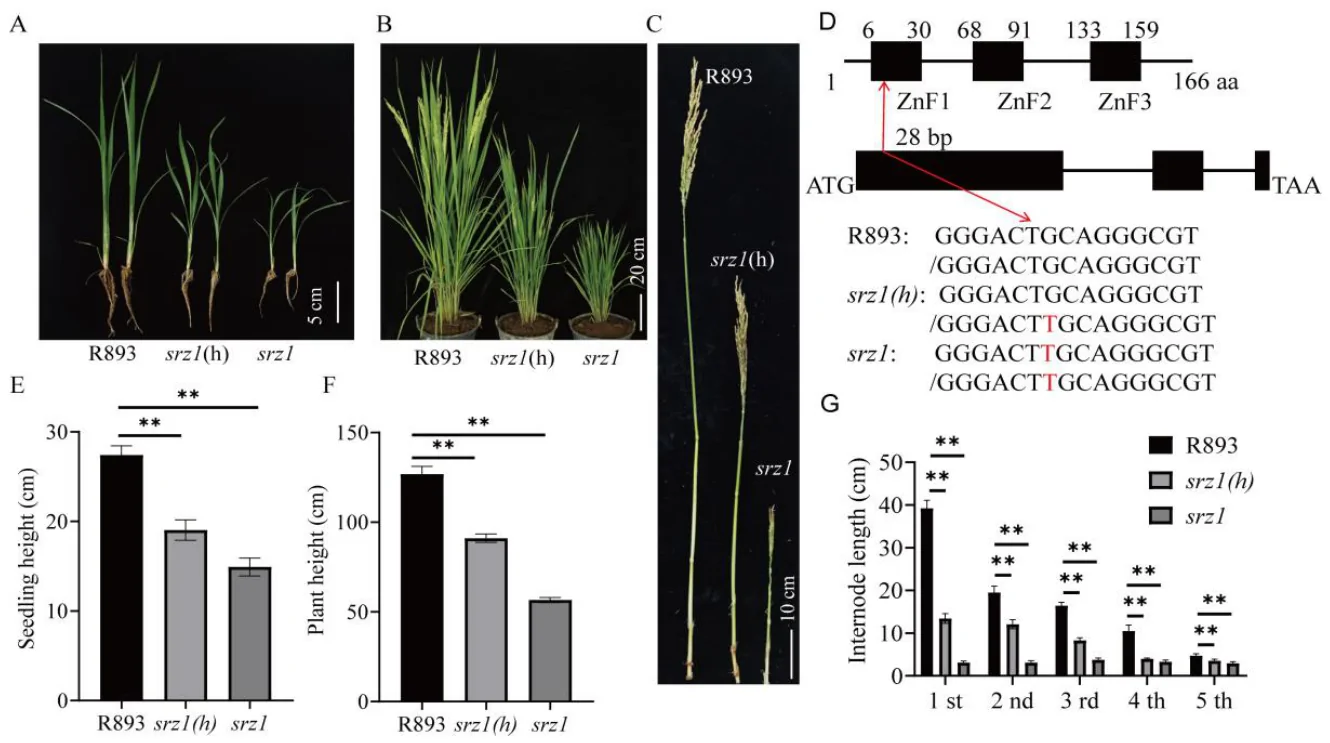
Phenotypic characterization of the *OsSRZ1* knockout mutant in rice. (**A**) Seedling phenotypes of wild-type (R893), homozygous knockout mutant (*srz1*), and heterozygous mutant (*srz1(h)*). (**B**) Plant phenotypes at heading stage of wild-type (R893), *srz1*, and *srz1(h)*. (**C**) Panicle-bearing stems (including five internodes and panicles) of wild-type (R893), *srz1*, and *srz1(h)*. (**D**) Mutation analysis of *OsSRZ1* in the *srz1* mutant. A single T nucleotide insertion was identified at 28 bp downstream of the ATG start codon, which falls within the first conserved motif. (**E**) Statistical analysis of plant height at seedling stage. (**F**) Statistical analysis of plant height at heading stage. (**G**) Statistical analysis of the lengths of individual internodes. Error bars represent SD. Asterisks indicate significant differences compared with the wild type (** *p* < 0.01, Student’s *t*-test).

To further investigate the biological functions of *SRZ* genes in rice, we selected *OsSRZ1* for gene editing using the CRISPR/Cas9 system. From the edited progeny, we obtained both heterozygous (*srz1(h)*) and homozygous (*srz1*) mutant lines (Figure 5A,D). Molecular characterization confirmed that the srz1 homozygous mutant carried a single T insertion at 28 bp downstream of the ATG start codon of *OsSRZ1*, whereas the *srz1(h)* line carried the T insertion in one allele (Figure 5D), which is located within the ZnF1 domain (GDWXCX_2−4_NX_6_CX_2_C motif). These mutant lines were then grown in the field for phenotypic analysis at multiple developmental stages. At the seedling stage (30-day-old seedlings), both *srz1(h)* and *srz1* mutants exhibited significantly reduced plant height compared with the wild-type R893 (Figure 5A,E). Notably, the *srz1(h)* line displayed an intermediate phenotype between the wild type and the *srz1* mutant, suggesting a dosage-dependent effect. At the heading stage, a similar phenotype was observed, with both mutant lines showing significantly reduced plant height and the *srz1(h)* line again showing a plant height intermediate between that of the wild type and the *srz1* mutant (Figure 5B,F).

To examine the contribution of individual internode shortening to the overall plant height reduction, we measured the lengths of all five stem internodes in the wild-type R893, the *srz1(h)* line, and the *srz1* mutant. Each internode was significantly shorter in both mutant lines compared with the wild type, with the *srz1(h)* line again showing internode lengths intermediate between those of the wild type and the *srz1* mutant (Figure 5C,G). Among the five internodes, the first internode below the panicle showed the greatest reduction in length, with the highest reduction ratio (Figure 5G).

Collectively, these results demonstrate that disruption of *OsSRZ1* leads to a significant reduction in plant height at both the seedling and heading stages. Further analysis revealed that this dwarf phenotype is attributable to the shortening of all five stem internodes, particularly in the first internode below the panicle. Notably, the heterozygous *srz1(h)* line consistently exhibited an intermediate phenotype between the wild-type R893 and the homozygous mutant across all measured traits, including plant height and internode length. These findings indicate that *OsSRZ1* regulates plant height by modulating internode elongation in rice, and that the mutation exhibits a dosage-dependent effect, suggesting that *OsSRZ1* exhibits incomplete dominance in controlling this agronomic trait.

### 3.7. Role of OsSRZ1 in Regulating Panicle Exsertion and Seed Setting Rate in Rice

**Figure 6:**
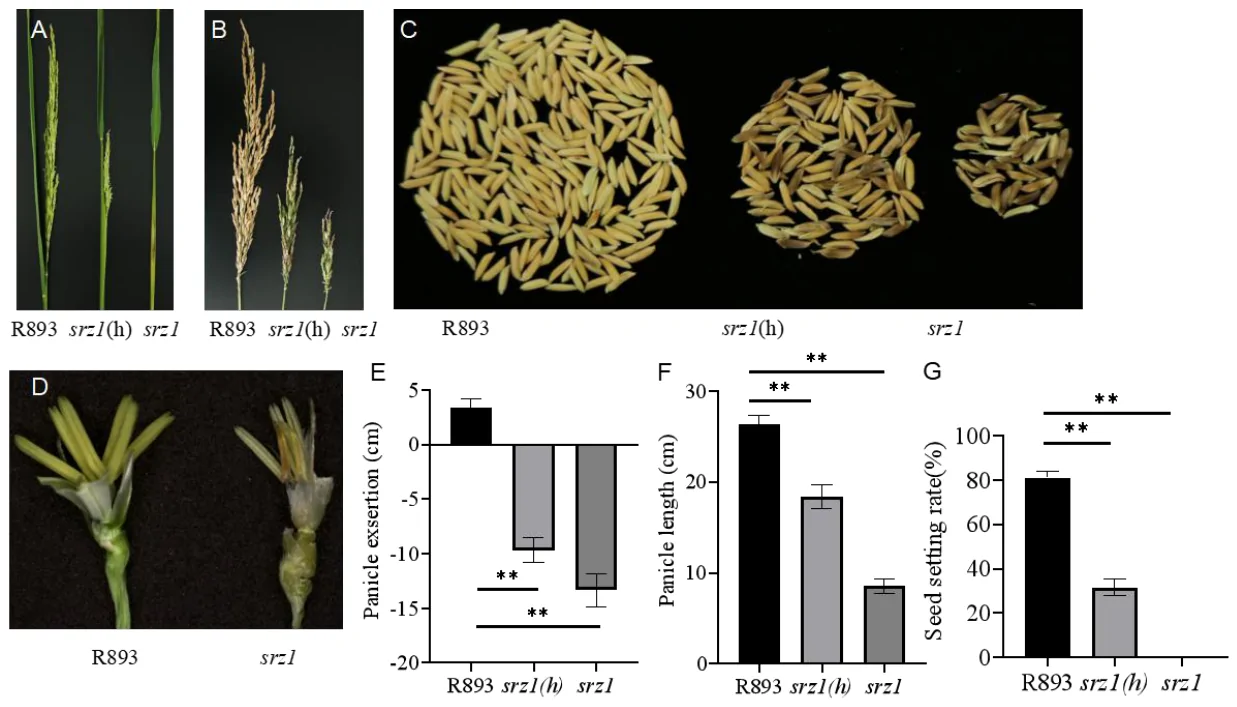
Phenotypic characterization of the panicle and spikelet traits of the *OsSRZ1* knockout mutant in rice. (**A**) Phenotypes of internodes enclosed by the leaf sheath in wild-type (R893), homozygous knockout mutant (*srz1*), and heterozygous mutant (*srz1(h)*). (**B**) Panicle phenotypes of wild-type (R893), *srz1*, and *srz1(h)*. (**C**) Seeds after threshing from a single panicle of wild-type (R893), *srz1*, and *srz1(h)*. (**D**) Anther phenotypes of wild-type (R893), *srz1*, and *srz1(h)*. (**E**) Analysis of the length of the panicle exsertion from the leaf sheath. (**F**) Analysis of panicle length. (**G**) Analysis of the seed setting rate (100%). Error bars represent SD (** *p* < 0.01, Student’s *t*-test).

To determine whether OsSRZ1 also affects reproductive development, we examined the panicle and spikelet phenotypes of wild-type R893, *srz1(h)*, and *srz1* at the heading stage, and analyzed the seed setting rate at maturity. Obvious defects in panicle sheath extrusion were observed in *OsSRZ1* mutant lines (Figure 6A). The panicles of *srz1* plants were completely enclosed by the leaf sheath and failed to exsert, whereas *srz1(h)* plants displayed an intermediate phenotype with partial panicle extrusion, which was significantly compromised compared with the wild type (Figure 6E). Consistent with the defective panicle emergence, mutations in *OsSRZ1* severely impaired panicle and floret development (Figure 6B,F). The *srz1* mutants produced the shortest panicles with obvious apical floret degeneration, while *srz1(h)* exhibited moderate panicle developmental defects between the wild type and *srz1*. Further cytological observation of mature florets showed that *srz1* displayed severely abnormal floral organ development and premature stamen senescence compared with R893 (Figure 6D). Phenotypic investigation of threshed single panicles revealed pronounced fertility defects in *OsSRZ1* mutants (Figure 6C). Statistical analysis demonstrated that the seed setting rate of *srz1(h)* was less than 40%, whereas *srz1* exhibited complete sterility with a seed setting rate of 0%, both significantly lower than that of R893 (Figure 6G).

Collectively, these results demonstrate that disruption of *OsSRZ1* leads to severe panicle enclosure, reduced panicle length, abnormal floret development, and a dramatic decrease in seed setting rate, indicating that *OsSRZ1* plays a critical role in regulating panicle exsertion and reproductive success in rice. Furthermore, the intermediate phenotype of *srz1*(h) across all measured reproductive traits suggests that *OsSRZ1* exhibits incomplete dominance in controlling these agronomic traits.

### 3.8. Role of OsSRZ1 in Regulating Pollen Fertility and PCD in Anthers

**Figure 7:**
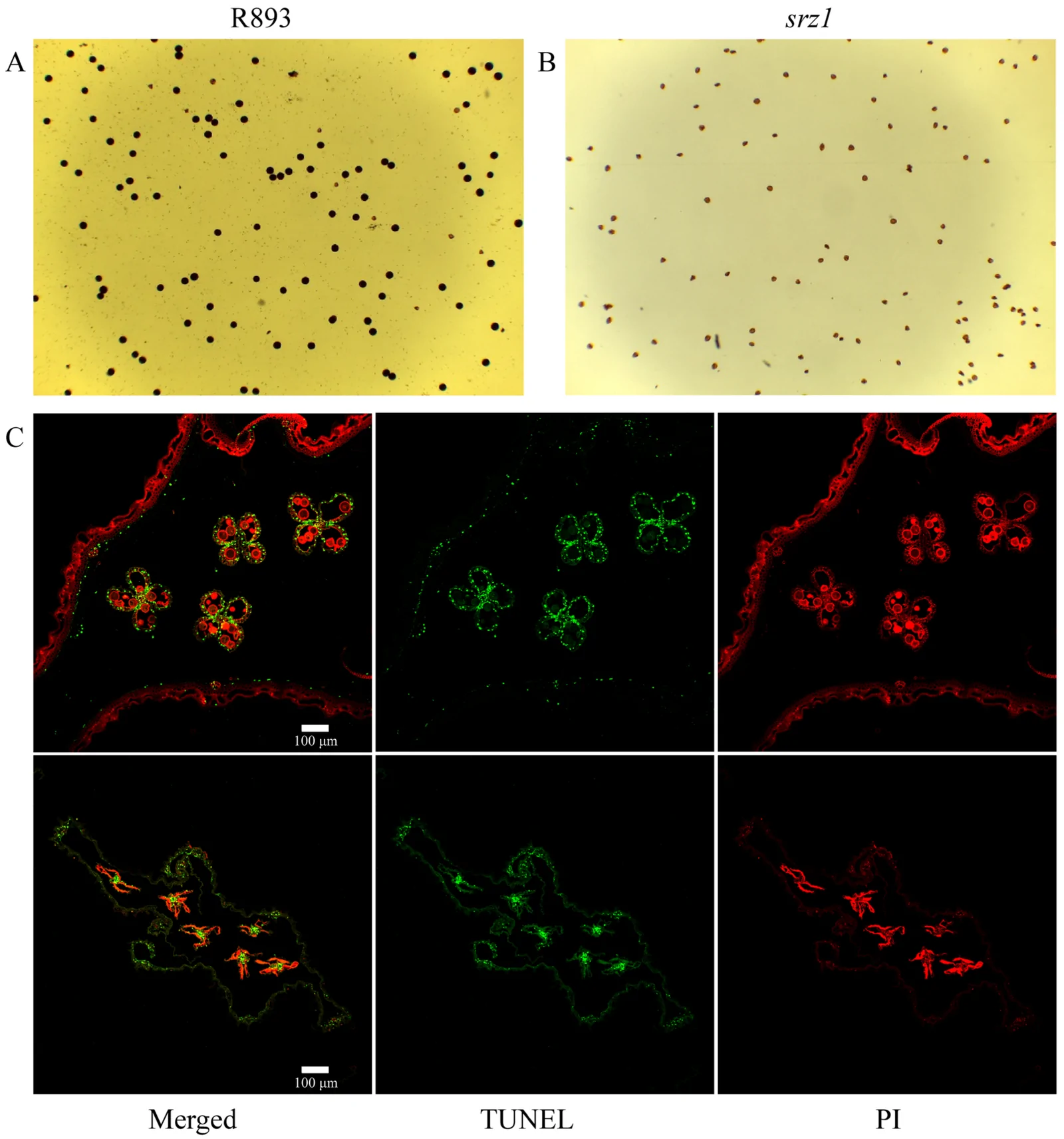
Pollen fertility and programmed cell death (PCD) analysis in R893 and *srz1*. (**A**) Microscopic observation of pollen fertility in R893. (**B**) Microscopic observation of pollen fertility in R893 and *srz1*. (**C**) TUNEL staining analysis of PCD in anthers of R893 and *srz1*. The red signal represents staining with propidium iodide (PI); yellow and green signals indicate TUNEL-positive nuclei of dead cells due to PCD.

Given the abnormal floret development and the complete sterility observed in the srz1 mutant, we examined pollen fertility and PCD in anthers of wild-type R893 and *srz1*. Microscopic observation showed that pollen grains of wild-type R893 were uniform in shape and fully stained, indicative of normal pollen maturation and full fertility (Figure 7A,B). In contrast, pollen from the *srz1* mutant displayed severe morphological malformations and failed to take up stain, resulting in complete male sterility, which demonstrates that loss of *OsSRZ1* function severely disturbs normal pollen development.

To determine whether the pollen abortion in *srz1* is associated with aberrant PCD during anther development, we performed TUNEL staining on anther sections of R893 and *srz1* (Figure 7C). R893 anthers exhibited a typical four-lobed structure with two pairs of pollen sacs, and PCD signals were detected specifically in the tapetum and middle layer at the corresponding developmental stages. In contrast, *srz1* anthers displayed severely disrupted lobe architecture and defective stratification of anther wall layers. Notably, all somatic wall layers, including the epidermis, endothecium, middle layer, and tapetum, underwent premature degeneration in *srz1*, which was associated with extensively activated premature PCD signals.

Taken together, these results indicate that *OsSRZ1* is essential for maintaining anther development and pollen fertility, as its mutation leads to premature PCD and complete male sterility.

## 4. Discussion

Increasingly frequent climate extremes have severely impacted plant growth, development, and productivity, particularly in crops [39]. SRZ proteins constitute a distinct subgroup of the RanBP2-type zinc finger family, characterized by the conserved GDWXCX_2−4_NX_6_CX_2_C motif. A previous preprint from our group identified 25 OsSRZ members in rice by including both the canonical GDWXCX_2−4_NX_6_CX_2_C motif and its sequence variants [29]. To better define the core structural features of the SRZ family and improve the reliability of subsequent analyses, we applied a more stringent criterion in the present study, retaining only proteins that strictly harbor the complete canonical motif and excluding those with degenerate or incomplete variants. Emerging evidence has implicated *SRZ* genes in various aspects of plant growth, development, and stress responses [20,21,26]. However, systematic characterization of this gene family remains relatively limited, and the biological functions of most SRZ members are poorly understood. In this study, we performed genome-wide identification of the *SRZ* gene family in rice and *Arabidopsis*, containing 14 and 10 members, respectively. We further analyzed their phylogenetic relationships, chromosomal distribution, gene structures, conserved motif compositions, promoter cis-regulatory elements, syntenic relationships, and spatiotemporal expression patterns. Additionally, we generated a CRISPR/Cas9-mediated knockout mutant of *OsSRZ1* in rice and found that *OsSRZ1* positively regulates plant height and internode elongation, acting as a incompletely dominant regulator of this trait. Our findings provide a comprehensive framework for understanding the structural evolution and functional divergence of the *SRZ* gene family and establish a solid foundation for future functional studies.

Phylogenetic classification and synteny analysis collectively revealed the evolutionary conservation and lineage-specific expansion of the plant *SRZ* gene family. The interspersed distribution of rice and *Arabidopsis* SRZ members into three distinct phylogenetic clades indicated that the diversification of the SRZ family occurred before the divergence of monocots and dicots, reflecting an ancient origin. However, such intermingled distribution of orthologous genes from distantly related species may also reflect phylogenomic discordance driven by pervasive ancient hybridization and incomplete lineage sorting during early angiosperm evolution [40]. Notably, different expansion patterns were observed between rice and *Arabidopsis SRZ* genes. In *Arabidopsis*, no tandem or segmental duplication events were detected, and all AtSRZ members exist as single-copy genes, suggesting that the *Arabidopsis* SRZ family has experienced limited expansion and its functional divergence is mainly driven by sequence variation rather than gene duplication. In contrast, rice *SRZ* genes underwent limited duplication events: one segmental duplication pair (*OsSRZ10* and *OsSRZ7*) and one tandem duplication pair (*OsSRZ8* and *OsSRZ9*) were identified, representing the only expansion events within the rice SRZ family. Their close phylogenetic relationships and conserved protein features further support their origins from recent duplication events. Collectively, these results indicate that although rice retained a small number of duplication-derived *SRZ* genes, the overall contribution of gene duplication to the expansion of the *SRZ* family is limited in both species, and that other mechanisms, such as retrotransposition or changes in regulatory sequences, may have played more prominent roles in shaping the functional diversity of this gene family. This finding mirrors the duplication patterns observed in other plant gene families, such as the MADS-box family, which exhibits lineage-specific transpositions and ancient tandem duplications [41], and the APETALA2 subfamily, which shows deep conservation alongside lineage-specific expansion patterns [42]. Furthermore, the conserved syntenic relationship between *OsSRZ8* and *AtSRZ4* demonstrates the retention of orthologous gene pairs across distant plant lineages, implying that these genes may preserve core biological functions. Such syntenic relationships have been shown to reliably identify orthologs for phylogenomics and comparative genomic analyses across the Brassicaceae and beyond [43]. Collectively, these evolutionary differences suggest that *SRZ* genes have undergone species-specific adaptive divergence, which may contribute to the functional divergence of developmental and stress regulatory pathways between monocots and dicots.

Exon–intron organization closely parallels phylogenetic relationships among SRZ members, suggesting divergent evolution from common ancestors, a pattern commonly observed in plant gene families [44,45]. Tandemly arrayed Cys-rich motifs with variable copy numbers and sequence heterogeneity have been associated with functional diversification in stress-related gene families [46]. The spatiotemporal expression patterns of *OsSRZ* genes exhibited marked tissue-preferential and stage-specific characteristics. Most members were predominantly expressed at reproductive and mature stages, suggesting functions primarily after vegetative growth. Notably, *OsSRZ2*, *OsSRZ3*, and *OsSRZ6* showed dominant accumulation in leaf blades, implying specialization in photosynthetic tissues, while *OsSRZ13* preferred leaf sheaths. Such divergent expression patterns are consistent with observations in other zinc finger gene families following gene duplication [47].

The types and numbers of cis-acting elements in promoter regions are closely linked to gene expression and functional specificity [48]. Notably, even single-nucleotide variations within cis-elements can lead to substantial phenotypic differences, as demonstrated by a W-box variant in the tomato *WRKY33* promoter that abolishes its cold-inducible self-transcription function [49]. Promoter analysis revealed abundant phytohormone-responsive and stress-related cis-elements, with total counts varying from 16 (*AtSRZ6*) to 55 (*AtSRZ7*) per member and stress-related elements exceeding one-third of all identified elements. This is consistent with previous observations that zinc finger protein gene promoters are typically enriched with stress- and hormone-responsive cis-elements [50,51].

RanBP2-type zinc finger proteins are known to participate in diverse biological processes, including vascular development, leaf polarity establishment, flowering time control, RNA editing, embryogenesis, ABA signaling, immunity, leaf senescence and Mg homeostasis [20,21,26,27,31]. However, the function and regulatory mechanisms of most SRZ members in rice remain largely unclear. To address this, we disrupted *OsSRZ1* using CRISPR/Cas9. The loss-of-function phenotypes of *OsSRZ1* provide direct genetic evidence for its role in regulating plant height in rice. The *srz1* and *srz1(h)* mutants exhibited a significant dwarf phenotype, indicating that OsSRZ1 regulates plant height in rice. Both heterozygous and homozygous mutants exhibited significant dwarfism with a clear dosage-dependent effect, indicating that *OsSRZ1* functions as a incompletely dominant gene in controlling internode elongation. This dosage sensitivity implies that the wild-type allele is required for full expression of the normal phenotype, and that the encoded protein likely operates in a concentration-dependent manner. Similar dosage effects have been observed in other rice zinc finger protein mutants [49,50]. The most pronounced reduction occurred in the first internode below the panicle of *srz1* and *srz1(h)*, suggesting that *OsSRZ1* may preferentially regulate cell elongation in specific stem tissues. A similar phenotype was also observed in the rice *hox12* mutant, where the uppermost internode was preferentially affected.

This internode-specific effect resembles the phenotype of mutants in which all internodes are shortened but the panicle neck internode shows the most severe reduction [52]. Stem internode elongation is regulated by multiple phytohormones, among which GA plays a predominant role. Notably, the *srz1* mutant phenotype also parallels that of *SLR1* gain-of-function mutants, which exhibit constitutive dwarfism due to the accumulation of DELLA proteins, the key suppressors of GA signaling [53]. This phenotypic similarity suggests that *OsSRZ1* mutation may compromise GA homeostasis, leading to reduced bioactive GA levels or impaired GA signaling output, and consequently resulting in the dwarf phenotype [54]. GA signaling is well established as the central pathway regulating internode elongation in rice, where DELLA protein SLR1 acts as a growth repressor [55,56,57]. GA triggers the degradation of SLR1 via the GID1-GID2 pathway, thereby relieving growth inhibition and promoting stem elongation [52,53]. In addition to GA, brassinosteroid (BR) signaling also plays a critical role in internode elongation; for instance, the DLT-OSH15 module regulates BR signaling and metabolism in an internode-specific manner [54]. Given that some SRZ members have been reported to function through RNA binding, we speculate that *OsSRZ1* may regulate internode elongation by associating with RNAs of key components in the GA pathway. Additionally, *OsSRZ1* is downregulated by multiple stresses, and its ectopic expression confers stress sensitivity [14]; it is also induced by ABA, JA, and H_2_O_2_ [29], suggesting a potential role in rice stress tolerance.

Notably, disruption of *OsSRZ1* led to a significant reduction in seed-setting rate, accompanied by abnormal elongation of panicles. This dual defect suggests that *OsSRZ1* functions in both reproductive success and inflorescence architecture. Microscopic observation revealed complete pollen abortion in *srz1* mutants, and TUNEL assays showed premature anther PCD. Similar regulatory connections between anther PCD, pollen viability, and seed-setting rate have been reported in other rice mutants. For instance, downregulation of the metallothionein gene *OsMT2b* resulted in elevated ROS levels and abnormal tapetum PCD dynamics, which subsequently led to sterile microspores and a severe reduction in seed-setting rate [58]. Likewise, mutations in *OsAMT1*, a DUF1771 domain-containing protein, caused premature tapetum PCD and reduced pollen fertility, accompanied by a dwarf phenotype with shortened internodes [59]. These studies reinforce the notion that proper spatiotemporal execution of PCD in the anther is critical for pollen development, anther dehiscence, and subsequent pollination, all of which directly influence fertilization and grain filling [60,61]. Together, these results suggest that OsSRZ1 regulates panicle elongation and anther PCD, and its disruption causes defective panicle exsertion and premature anther PCD, collectively leading to reduced seed-setting.

## 5. Conclusions

Here, we systematically identified 14 *SRZ* genes in rice and 10 in *Arabidopsis*. Comprehensive characterization revealed their evolutionary conservation, limited lineage-specific duplication, and abundant hormone- and stress-responsive cis-elements. Most rice *SRZ* genes were preferentially expressed in reproductive and mature tissues, indicating functional divergence and potential roles in post-vegetative development. CRISPR/Cas9-mediated knockout of *OsSRZ1* revealed that it positively regulates plant height and internode elongation in a dosage-dependent manner, while its loss of function caused panicle enclosure, premature anther PCD, and complete pollen abortion, resulting in 0% seed setting in *srz1* mutants. *OsSRZ1* is a pivotal pleiotropic regulator of rice plant architecture and reproductive fertility, providing a fundamental framework for future studies of the SRZ family.

## Figures and Tables

**Table 1:** Primers used in this study.

Primer	Forward Primer Sequence (5′ → 3′)	Reverse Primer Sequence (5′ → 3′)
T1	GGCAGCCAGGAGACTGGGACTGC	AAACGCAGTCCCAGTCTCCTGGCT
T2	GCCGTCTATGTTTGATCACAGGTC	AAACGACCTGTGATCAAACATAGA
F-U/R-gRNA	CTCCGTTTTACCTGTGGAATCG	CGGAGGAAAATTCCATCCAC
F-T1/R-T2	GGCAGCCAGGAGACTGGGACTGC	AAACGACCTGTGATCAAACATAGA
F-B1	TTCAGAGGTCTCTCTCGACCTGGAATCGGCAGCAAAGG	
B2-R	AGCGTGGGTCTCGTGCAGGGTCCATCCACTCCAAGCTC	
F-B2	TTCAGAGGTCTCTCTGACACTGGGAATCGGCAGCAAAGG	
R-BL	AGCGTGGGTCTCTCTGACGGGTCCATCCACTCCAAGCTC	
*SRZ1*-F/*SRZ1*-R	AGCTCACCAACACAGCAG	TTCAAAAGTCCGTAACTACCA

**Table 2:** SRZ family members identified in rice and *Arabidopsis*.

Gene	Gene ID	Chr	Protein Length	Function	Other Designation
*OsSRZ1*	LOC_Os02g10920	2	173	Regulate leaf senescence and respond to multiple stresses [30]	*LS1*
*OsSRZ2*	LOC_Os06g04920	6	145		
*OsSRZ3*	LOC_Os01g37460	1	139	Maintain Mg homeostasis and regulate leaf development [31]	*OsRZF1*; *LMGC1*
*OsSRZ4*	LOC_Os04g02000	4	343		
*OsSRZ5*	LOC_Os01g07070	1	500		
*OsSRZ6*	LOC_Os01g59980	1	950		
*OsSRZ7*	LOC_Os02g17470	2	471		
*OsSRZ8*	LOC_Os03g50120	3	504		
*OsSRZ9*	LOC_Os03g50430	3	523		
*OsSRZ10*	LOC_Os06g22700	6	318		
*OsSRZ11*	LOC_Os07g19560	7	394		
*OsSRZ12*	LOC_Os07g22024	7	647		
*OsSRZ13*	LOC_Os07g30820	7	288		
*OsSRZ14*	LOC_Os09g12800	9	479		
*AtSRZ1*	AT3G15680	3	164	Represses phloem development; modulates leaf adaxial–abaxial polarity [20,21]	*JUL1*; *JULGI1*
*AtSRZ2*	AT5G25490	5	170	Represses phloem development; mediates light-dependent root growth signaling [20]	*JUL2*; *JULGI2*
*AtSRZ3*	AT2G26695	2	138		
*AtSRZ4*	AT1G48570	1	455		
*AtSRZ5*	AT2G17975	2	268	Negatively regulates ABA signaling [22]	*SRP1*
*AtSRZ6*	AT1G55040	1	849	Essential for embryogenesis [25]	*OZ2*; *SED1*
*AtSRZ7*	AT1G70650	1	466		
*AtSRZ8*	AT4G28990	4	347		
*AtSRZ9*	AT5G17790	5	758	Critical for chloroplast RNA editing [13,23,24]	*OZ1*; *VAR3*
*AtSRZ10*	AT5G58470	5	422	Promotes flowering; regulates immunity [26,27,28]	*TAF15b*

## Data Availability

The original contributions presented in this study are included in the article; further inquiries can be directed to the corresponding author.
